# *PLS3* Mutations Cause Severe Age and Sex-Related Spinal Pathology

**DOI:** 10.3389/fendo.2020.00393

**Published:** 2020-06-23

**Authors:** Riikka E. Mäkitie, Tuukka Niinimäki, Maria Suo-Palosaari, Anders Kämpe, Alice Costantini, Sanna Toiviainen-Salo, Jaakko Niinimäki, Outi Mäkitie

**Affiliations:** ^1^Folkhälsan Institute of Genetics, Helsinki, Finland; ^2^Research Program for Clinical and Molecular Metabolism, Faculty of Medicine, University of Helsinki, Helsinki, Finland; ^3^Molecular Endocrinology Laboratory, Department of Metabolism, Digestion and Reproduction, Imperial College London, London, United Kingdom; ^4^Department of Surgery, Oulu University Hospital, Oulu, Finland; ^5^Research Unit of Medical Imaging, Physics and Technology, University of Oulu, Oulu, Finland; ^6^Medical Research Center, Oulu University Hospital, University of Oulu, Oulu, Finland; ^7^Department of Molecular Medicine and Surgery, Center for Molecular Medicine, Karolinska Institutet, and Clinical Genetics, Karolinska University Hospital, Stockholm, Sweden; ^8^Department of Pediatric Radiology, Medical Imaging Center, University of Helsinki and Helsinki University Hospital, Helsinki, Finland; ^9^Children's Hospital and Pediatric Research Center, University of Helsinki and Helsinki University Hospital, Helsinki, Finland

**Keywords:** PLS3, magnetic resonance imaging, vertebral compression fracture, schmorl node, intervertebral disc

## Abstract

**Objective:** Mutations in the X-chromosomal *PLS3-*gene, encoding Plastin 3, lead to severe early-onset osteoporosis, suggesting a major role for PLS3 in bone metabolism. However, the consequences of abnormal PLS3 function in bone and other tissues remain incompletely characterized. This study evaluated spinal consequences of aberrant PLS3 function in patients with *PLS3* mutations.

**Design:** A cross-sectional cohort study with spinal magnetic resonance imaging of 15 *PLS3* mutation-positive (age range 9–77 years) and 13 mutation-negative (9–70 years) subjects. Images were reviewed for spinal alignment, vertebral heights and morphology, intervertebral disc changes and possible endplate deterioration.

**Results:** Vertebral changes were significantly more prevalent in the mutation-positive subjects compared with the mutation-negative subjects; they were most abundant in upper thoracic spine, and in all age groups and both sexes, although more prominent in males. Difference in anterior vertebral height reduction was most significant in T5 and T6 (*p* = 0.046 and *p* = 0.041, respectively). Mid-vertebral height reduction was most significant in T3 and T5 (*p* = 0.037 and *p* = 0.005, respectively), and, for male mutation-positive subjects only, in T4 and T6–10 (*p* = 0.005–0.030 for each vertebra). Most of the abnormal vertebrae were biconcave in shape but thoracic kyphosis or lumbar lordosis were unchanged. Vertebral endplates were well-preserved in the mutation-positive subjects with even fewer Schmorl nodes than the mutation-negative subjects (10 vs. 16).

**Conclusions:** Compromised PLS3 function introduces severe and progressive changes to spinal structures that are present already in childhood, in both sexes and most abundant in upper thoracic spine. Cartilaginous structures are well-preserved.

## Introduction

Compromised bone strength predisposes to vertebral compression fractures (VCFs) and changes in spinal alignment. VCFs are the most common complication of osteoporosis and spinal pathology a primary finding in different forms of bone fragility disorders, such as osteogenesis imperfecta (OI), occurring in up to 78% of OI patients (1). Due to skeletal fragility and ligamentous laxity, OI patients also suffer from other spinal changes, such as kyphosis, scoliosis, basilar impression and lumbosacral pathology (2). VCFs and other spinal abnormalities are linked to the disease severity and age of onset and can be greatly debilitating, resulting in severe disfigurement, chronic pain and decreased quality of life (3).

PLS3 osteoporosis was first described in 2013 as mutations in the X-chromosomal *PLS3*, encoding Plastin 3 (PLS3), were recognized to lead to severe childhood-onset osteoporosis (4). Since then, many families worldwide have been reported with variable forms of PLS3-related skeletal fragility (5–8). Due to its X-chromosomal inheritance pattern, hemizygous males are typically more severely affected than heterozygous females, portraying markedly decreased bone mineral density (BMD) and multiple peripheral and VCFs starting from an early age (5, 6). Heterozygous females present with a variable phenotype ranging from normal to skeletal fragility with few or multiple fractures (5, 6, 8). Bone tissue is characterized by low bone turnover with reduced osteoblast and osteoclast numbers and disturbed matrix mineralization (6, 7, 9).

PLS3 is thought to function as a modulator of intracellular cytoskeletal organization and actin bundling, thus partaking in various cellular functions such as cell migration and adhesion (10). In bone, PLS3 is proposed to regulate osteocyte function, their mechanosensing properties and matrix mineralization (7), while studies on *Pls3* knock-out (KO) mice also point to an osteoclast-regulatory role (11). However, the exact pathomechanisms of impaired PLS3 function in bone are still unknown and hence the skeletal features, natural course and optimal treatment of PLS3 osteoporosis remain incompletely characterized. Furthermore, although no extra-skeletal manifestations are evident in humans or in *Pls3*-KO mice, the importance of PLS3 in other tissues remains elusive (4, 5, 11).

To date, we have identified four unrelated Finnish families with PLS3 osteoporosis due to different *PLS3* mutations (5, 8, 12). Granted the access to this unique cohort of *PLS3* mutation-positive subjects, we set out to evaluate their spinal pathology using magnetic resonance imaging (MRI). Deduced from observed VCFs in plain radiographs, thoracic kyphosis and anamnestic loss of adult height (5), we hypothesized that spinal changes are a particular feature of PLS3 osteoporosis and that their age of onset, severity and location might differ from that of other forms of osteoporosis (13, 14). Specifically, we evaluated the number, location and severity of affected vertebrae, abnormalities in spinal stature and possible changes in cartilaginous tissues. Our findings on 15 mutation-positive subjects indicate that impaired PLS3 function causes significant early-onset and progressive changes in the spine in both males and females.

## Subjects and Methods

### Subjects

**Figure 1:**
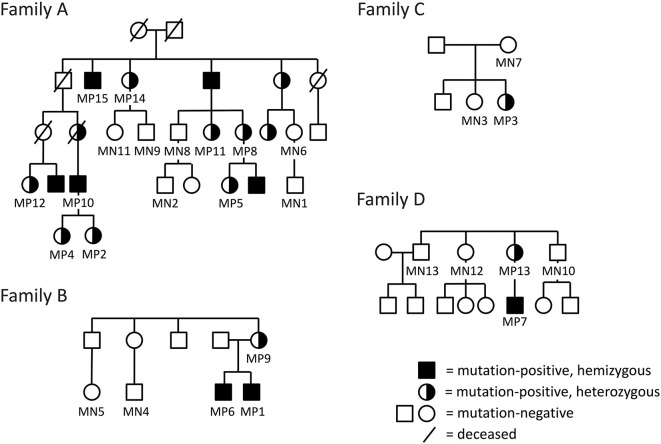
Pedigrees of the four included Finnish families with *PLS3* mutations; **(A)** intronic splice site mutation c.73–24T>A (p.Asp25Alafs*17) **(B)**, intragenic tandem duplication within *PLS3* **(C)**, *de novo* heterozygous missense mutation c. 1424A>G (p.Asn446Ser) **(D)**, non-sense mutation c.766C>T (p.Arg256*). The pedigrees have been modified to ensure anonymity. Squares represent males, circles females.

We have previously identified four unrelated Finnish families with PLS3 osteoporosis due to different *PLS3* mutations (Figure 1) (5, 8, 12). Family A has an intronic *PLS3* splice site mutation c.73–24T>A (p.Asp25Alafs^*^17) as previously described (5). Family B has a 12.5 kb tandem duplication spanning intron 2 to 3 of *PLS3*, identified by array-comparative genomic hybridization and predicted to lead to loss of function (12). Family C includes a single subject with a *de novo* heterozygous missense mutation c.1424A>G (p.Asn446Ser), identified through Sanger sequencing of the whole gene (8). Lastly, Family D has a nonsense mutation c.766C>T (p.Arg256^*^), also found by Sanger sequencing (8). In addition to these identified individuals, we have offered genetic testing to relatives at risk and as a result, to date, altogether 21 *PLS3* mutation-positive subjects have been identified in these four families.

For the current study, we offered all mutation-positive subjects (*n* = 20, one deceased) the opportunity to participate in a study concerning spinal health in PLS3 osteoporosis. Altogether 15 mutation-positive subjects complied. To form a comparable control group, we offered participation to also mutation-negative family members (*n* = 18) and of them, 13 individuals consented. In addition, we used previously obtained spinal MR-images of a *WNT1* mutation-positive subject for comparison; these images were originally obtained for our analysis of WNT1-associated spinal pathology (14). All subjects or their guardians gave a written informed consent upon participation in the study. The research protocol was approved by the Research Ethics Board of Helsinki University Hospital.

### Genetic Evaluations

We performed genetic validations on DNA extracted from peripheral blood, as previously described (5, 8, 12). We screened all samples for the families' pertinent, previously identified *PLS3* mutation (NCBI Reference Sequence NM_005032).

### Clinical Cohort Characteristics

Altogether 11/15 of the mutation-positive subjects were clinically evaluated during a study visit at Helsinki University Hospital for clinical features, including anthropometry, spinal alignment and spinal abnormalities. For all subjects, data on sustained fractures and previous medical and surgical treatments were collected by patient interviews and from prior medical records.

### Magnetic Resonance Imaging

MR-imaging of the thoracic and lumbar spine were performed in 2018 at different medical healthcare providers in Finland. All images were obtained with one of three different 1.5-T MRI scanners (Optima MR360 and Signa HDxt; General Electric Healthcare, and MAGNETOM ESSENZA; Siemens Healthcare) using a spine coil. The imaging sequences were selected from clinical protocols including T1-weighted sagittal images (TR/TE 470–800/10–11 ms, FOV 280–330 mm, in-plane resolution 0.6–0.9 × 0.6–0.9 mm, slice thickness 3–4 mm) and T2-weighted sagittal images (TR/TE 2,600–3,500/80–98 ms, FOV 280–330 mm, in-plane resolution 0.6–0.9 x 0.6–0.9 mm, slice thickness 3–5 mm) and T2-weighted fat-saturated inversion recovery images (TR/TI/TE 3,000–3,200/130–160/47–67, FOV 310–320 mm, in-plane resolution 1.3–1.4 × 1.3–1.4 mm, slice thickness 3–4 mm) of lumbar and thoracic spine. All images were obtained in supine position with the subject lying down, legs extended horizontally.

The MRIs were first independently assessed for spinal changes by an orthopedic surgeon and three experienced radiologists who were blinded to the subjects' genotype and phenotype. Statistical calculations for the vertebral measurements were made from median values of two of the reviewers' (MSP and STS) independent calculations. The final conclusions were evaluated for discrepancy by calculating Intraclass Correlation Coefficient (ICC) for 10 randomly selected cases; for all the cases the ICC score was >0.80 and therefore the analyses were considered highly consistent and reproducible. The MRI data were assessed as described later. Detailed methods for measuring these listed parameters are available on request from the authors.

#### Vertebral Morphology

Changes were evaluated separately for each thoracic and lumbar vertebral body (T1 to L4) using different clinically accepted classification methods. To analyze the thoracic and lumbar vertebral morphology, anterior, mid-vertebral and posterior heights of the vertebrae as well as the anterior–posterior length at the level of the basivertebral vein were measured. Vertebral height loss was measured from ratios of anterior/posterior and mid-vertebral/posterior heights. In addition, the ratio of mid-vertebral height to anterior–posterior length (mid-vertebral height/depth ratio) was assessed. We used a previously described classification system for descriptive assessment of vertebral morphology (15). The method classifies vertebral changes in three subgroups according to type of deformity: grade 1 for normal variation, grade 2 for anterior wedging (anterior height reduction a ≥20 or b ≥50%), and grade 3 for compression deformity (mid-vertebral height reduction a ≥20 or b ≥30%) (15). The measures of mid-vertebral height/depth ratios were compared between the mutation-positive and -negative groups.

#### Endplates

The number, location and extent of Schmorl nodes (SN) were recorded using a modified classification system introduced by Samartzis et al. (16). The recorded domains for each SN were vertebrae level (T4–L4), endplate involvement (rostral, caudal, or both) and size (<1/3, <2/3, or >2/3 of endplate).

#### Intervertebral Discs

Changes in the intervertebral discs were assessed by measuring the total surface areas of the intervertebral discs from L3 to L5 and visually from images.

#### Spinal Alignment

Changes in spinal stature were evaluated by calculating degrees for thoracic kyphosis and lumbar lordosis. The angles were calculated using a modified Cobb's method by drawing tangent lines to selected vertebral bodies (T1 and T12 for thoracic spine and L1 and L5 for lumbar spine) (17). Median values were used to compare differences between the two groups.

### Statistical Analysis

Descriptive data are reported as median and range. Normality of the data was assessed using Kolmogorov–Smirnov and Shapiro–Wilk and visually using histograms. Mann–Whitney *U*-test was used as appropriate (SPSS Statistics 25; IBM Corporation, Armonk, NY, USA). A *p* < 0.050 was considered statistically significant.

## Results

### Cohort Characteristics

#### Mutation-Positive Subjects

The 15 *PLS3* mutation-positive subjects (10 females, 5 males) ranged in age from 9 to 77 years (median 42 years) (Figure 1, Table 1). Altogether eight (53%) of them had sustained at least one previous long-bone fracture and seven (47%) at least one VCF. Seven subjects (47%) reported back pain; three of them considered the pain disabling. Two of the nine adults reported height loss of ≥4 cm. Seven subjects had received osteoporosis medication prior to the study and for two of them, the treatment was still ongoing at the time of the study (Table 1).

**Table 1:** History of fractures and previous osteoporosis medication for 15 subjects with a hemizygous/heterozygous *PLS3* mutation and 13 mutation-negative subjects.

**Code**	**Sex (F/M)**	***PLS3*** **mutation**	**Age category** **(years)**	**Osteoporosis medication**	**Peripheral fractures**		**History of VCFs**	**Back pain**	**Height loss**
					**Number**	**Age at 1st fracture (years)**			
**Mutation-positive subjects:** 15 subjects, 10 females/5 males, age range 9–77 years, median 42 years									
**Mutation-negative subjects:** 13 subjects, 6 females/7 males, age range 9–70 years, median 40 years									
**Mutation-positive subjects**									
MP1	M	2	0–11	None	2	5	No	No	N/A
MP2	F	1	12–20	None	–	–	No	N/A	N/A
MP3	F	3	12–20	PAM 2013, ZOL 2014–	5	2	Yes	Yes	N/A
MP4	F	1	12–20	None	–	–	No	N/A	N/A
MP5	F	1	12–20	None	–	–	No	No	N/A
MP6	M	2	21–40	ZOL 2007–2009	1	17	Yes	No	N/A
MP7	M	4	21–40	None	4	10	Yes	Yes	N/A
MP8	F	1	41–60	ZOL 2016–	–	–	No	Yes	No
MP9	F	2	41–60	None	–	–	No	Yes	1 cm
MP10	M	1	41–60	ZOL −2011	10	21	Yes	Yes	3 cm
MP11	F	1	41–60	None	–	–	No	N/A	N/A
MP12	F	1	41–60	PTH 2011–2013	–	–	Yes	No	No
MP13	F	4	41–60	None	1	35	No	No	2 cm
MP14	F	1	61–80	PTH 2007–2008 ZOL 2010–2014	>10	5	Yes	Yes	5 cm
MP15	M	1	61–80	ZOL 4 years	4	55	Yes	Yes	11 cm
**Mutation-negative subjects**									
MN1	M	None	0–11	None	1	6	No	N/A	N/A
MN2	M	None	0–11	None	–	–	No	N/A	N/A
MN3	F	None	12–20	None	1	9	No	N/A	N/A
MN4	M	None	12–20	None	–	–	No	N/A	N/A
MN5	F	None	12–20	None	1	2	No	N/A	N/A
MN6	F	None	21–40	None	2	16	No	N/A	N/A
MN7	F	None	21–40	None	8	1	No	N/A	N/A
MN8	M	None	41–60	None	2	16	No	No	N
MN9	M	None	41–60	DMAB 2 years	3	30	Yes	Yes	N
MN10	M	None	41–60	None	1	17	No	N/A	N/A
MN11	F	None	41–60	None	1	8	No	Yes	N
MN12	F	None	61–80	None	2	59	No	N/A	N/A
MN13	M	None	61–80	ALN 2002–2007	–	–	Yes	N/A	3 cm

PAM, pamidronate; ZOL, zoledronic acid; PTH teriparatide; DMAB, denosumab; ALN, alendronate. F, female; M, male; N/A, data not available; VCF, vertebral compression fracture.

PLS3 mutations: 1. heterozygous/hemizygous deletion c.73–24T>A (p.Asp25Alafs^*^17); 2. heterozygous/hemizygous duplication spanning intron 2 to 3; 3. heterozygous missense mutation c.1424A>G (p.Asn446Ser); 4. heterozygous/hemizygous nonsense mutation c.766C>T (p.Arg256^*^).

#### Mutation-Negative Subjects

The 13 *PLS3* mutation-negative subjects (6 females, 7 males) ranged in age from 9 to 70 years (median 40 years) (Figure 1, Table 1). Altogether 10 (71%) of them had sustained one or more previous peripheral fractures, two had previously been diagnosed with compression fractures, two had mild back pain and none reported a ≥4 cm height loss. Two had received osteoporosis medications prior to the study.

### MRI Findings

#### Vertebrae

**Figure 2:**
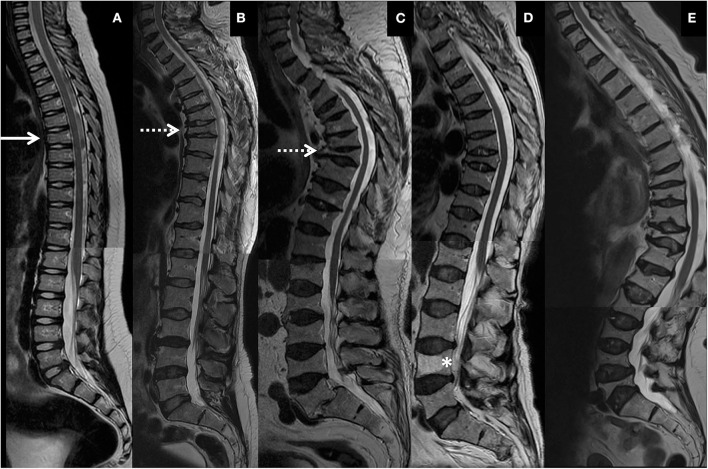
Representative spinal magnetic resonance images of thoracic and lumbar spines of four *PLS3* mutation-positive subjects. **(A)** 9-year-old male, **(B)** 46-year-old male, **(C)** 77-year-old male, and **(D)** 70-year-old female. Images show mild vertebral changes in childhood (solid arrow), multiple and severe vertebral compression fractures and kyphosis in early adulthood (dotted arrows), progression of spinal changes with increasing age. **(D)** portrays vertebral changes in heterozygous females and a vertebral hemangioma (asterisk). Subject in **(A)** harbors an intragenic tandem duplication within *PLS3*, and subjects in **(B–D)** a heterozygous/hemizygous *PLS3* deletion c.73–24T>A (p.Asp25Alafs*17). **(E)** Shows thoracic and lumbar spine images of a 74-year-old female with WNT1 osteoporosis due to a heterozygous *WNT1* mutation p.Cys218Gly, portraying the differences between PLS3 and WNT1 osteoporosis in type and location of vertebral compression fractures, changes in spinal stature and presence of simultaneous deterioration in cartilaginous tissues. The images have been combined from separate MR-images of thoracic and lumbar spines.

**Figure 3:**
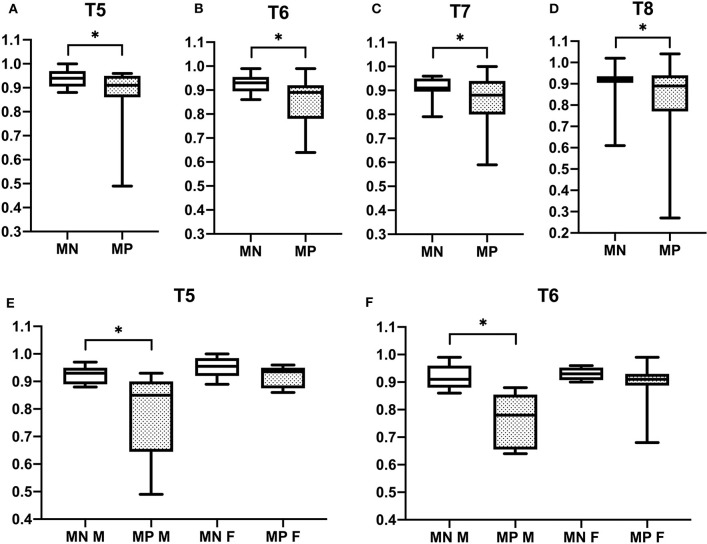
Comparison of anterior vertebral height reduction in 15 *PLS3* mutation-positive and 13 mutation-negative subjects. Ratios of anterior to posterior vertebral height in **(A)** T5, **(B)** T6, **(C)** T7, **(D)** T8, **(E)** T5, and **(F)** T6. MP = mutation-positive, MN, mutation-negative; M, male; F, female. The *PLS3* mutation-positive subjects harbor different mutations: nine with heterozygous/hemizygous deletion c.73–24T>A (p.Asp25Alafs*17), three with heterozygous/hemizygous intragenic tandem duplication within *PLS3*, one with heterozygous missense mutation c.1424A>G (p.Asn446Ser), and two with heterozygous/hemizygous non-sense mutation c.766C>T (p.Arg256*). *p < 0.05.

**Figure 4:**
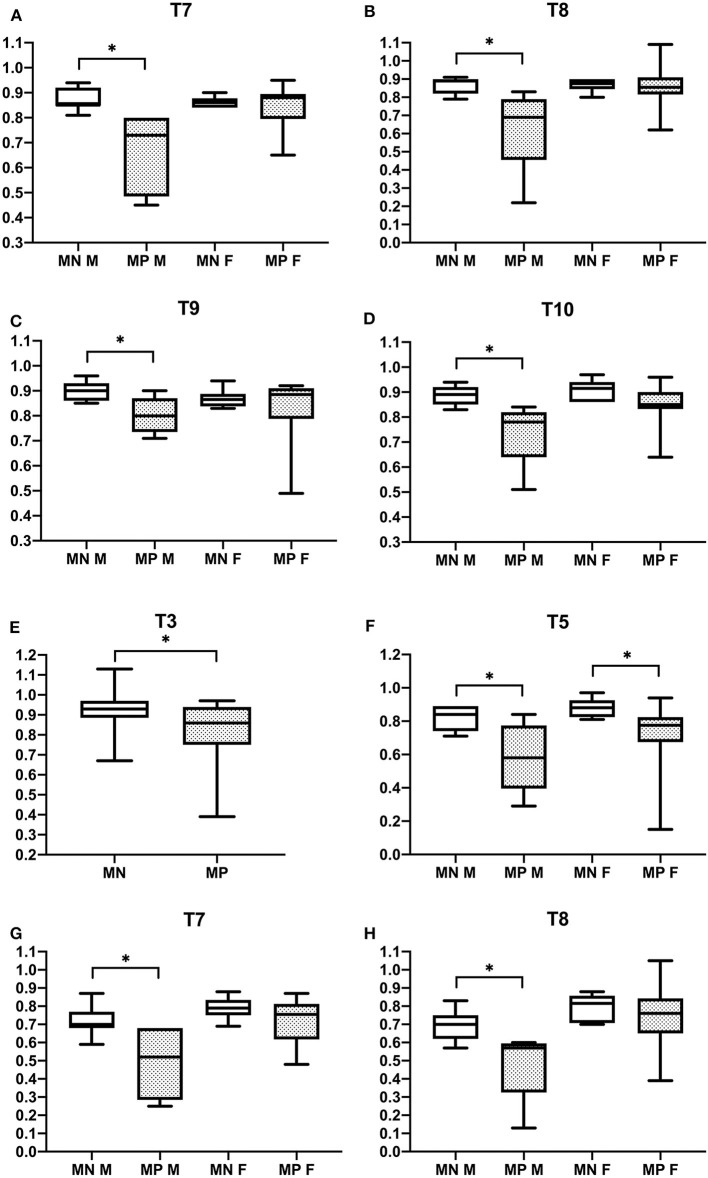
Comparison of ratios of mid-vertebral to posterior vertebral height **(A–D)** and mid-vertebral height to anterior–posterior length **(E–H)** in 15 *PLS3* mutation-positive and 13 mutation-negative subjects. Ratios of mid-vertebral to posterior vertebral height in **(A)** T7, **(B)** T8, **(C)** T9, and **(D)** T10 and ratios of mid-vertebral height to anterior–posterior length in **(E)** T3, **(F)** T5, **(G)** T7, and **(H)** T8. MP, mutation-positive, MN, mutation-negative, M, male, F, female. The *PLS3* mutation-positive subjects harbor different mutations: nine with heterozygous/hemizygous deletion c.73–24T>A (p.Asp25Alafs*17), three with heterozygous/hemizygous intragenic tandem duplication within *PLS3*, one with heterozygous missense mutation c.1424A>G (p.Asn446Ser), and two with heterozygous/hemizygous nonsense mutation c.766C>T (p.Arg256*). *p < 0.05.

**Figure 5:**
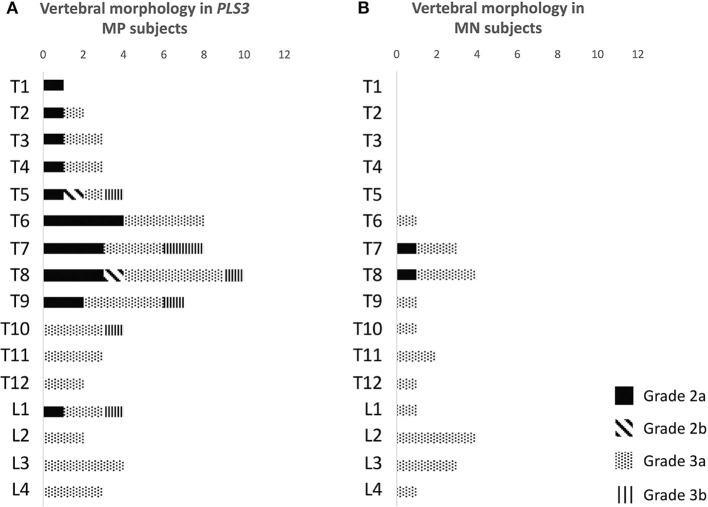
Classification of abnormal vertebrae in **(A)** 15 *PLS3* mutation-positive and **(B)** 13 mutation-negative subjects. Each abnormal vertebra was classified according to a classification system for descriptive assessment of vertebral morphology, introduced by Mäkitie et al. (15), according to type of deformity: grade 1 for normal variation, grade 2 for anterior wedging (anterior height reduction *a* ≥20% or *b* ≥50%), and grade 3 for compression deformity (mid-vertebral height reduction a ≥20% or b ≥30%) (15). MP, mutation-positive; MN, mutation-negative The *PLS3* mutation-positive subjects harbor different mutations: nine with heterozygous/hemizygous deletion c.73–24T>A (p.Asp25Alafs*17), three with heterozygous/hemizygous intragenic tandem duplication within *PLS3*, one with heterozygous missense mutation c.1424A>G (p.Asn446Ser), and two with heterozygous/hemizygous non-sense mutation c.766C>T (p.Arg256*).

Vertebral changes were significantly more frequent in the mutation-positive subjects compared with the mutation-negative subjects. They were present already in children and increasingly prevalent with age. These vertebral changes were most abundant and marked in the upper thoracic spine, specifically in T3–T8 (Figures 2–4, Table 2). Loss of anterior vertebral height was most significant in T5 and T6 in all mutation-positive subjects compared with mutation-negative subjects (*p* = 0.046 and *p* = 0.041, respectively) (Figure 3). For male subjects only, the difference was significant in T5–T8 (*p* = 0.030 for T5 and *p* = 0.010 for others). In females, corresponding differences did not reach statistical significance. Loss of mid-vertebral height was most significant in T10 (*p* = 0.004) for all mutation-positive subjects but for male subjects, the difference was significant in T4 and T6–T10 (*p* = 0.030, *p* = 0.018, *p* = 0.003, *p* = 0.010, *p* = 0.010, and *p* = 0.005, respectively) (Figure 4). In females, the difference was significant in T12 (*p* = 0.031). When comparing the ratio of mid-vertebral height to anterior–posterior length, the difference between all the mutation-positive and -negative subjects was significant in T3 and T5 (*p* = 0.037 and *p* = 0.005, respectively) (Figure 4). For male subjects alone, the difference was significant in T5, T7, and T8 (*p* = 0.018, *p* = 0.030, and *p* = 0.018, respectively), and in females, the difference was significant in T5 (*p* = 0.016) (Figure 4). Significant differences persisted when results were compared in only subjects under the age of 30 years: decrease in anterior height was significant in T6 (*p* = 0.022) and decrease in mid-vertebral height in T10 (*p* = 0.002). In the mutation-positive subjects, most of the abnormal vertebrae were both reduced in height and biconcave in shape and graded as 3a according to the Mäkitie et al. vertebral morphology classification (Figure 5). None of the abnormal vertebrae protruded into the spinal canal or caused evident spinal stenosis.

**Table 2:** *p*-values for differences in vertebral measurements between 15 subjects with a hemizygous/heterozygous *PLS3* mutation and 13 mutation-negative subjects.

**Vertebra**	**All** **subjects**	**Change in mean** **(MP vs. MN)**	**M**	**F**	**All** **subjects**	**Change in mean** **(MP vs. MN)**	**M**	**F**	**All subjects**	**Change in mean** **(MP vs. MN)**	**M**	**F**
	**Anterior–posterior height ratio**				**Mid-vertebral–posterior height ratio**				**Mid-vertebral height–AP-length ratio**			
Th1	0.717	0.005	1.000	0.428	0.339	−0.018	0.432	0.792	0.235	−0.061	1.000	0.220
Th2	0.052	0.021	0.639	0.220	1.000	−0.005	0.755	0.792	0.156	−0.067	0.639	0.056
Th3	0.892	−0.011	0.343	0.792	0.185	−0.048	0.530	0.313	**0.037**	−0.102	0.106	0.093
Th4	0.964	−0.002	0.149	0.875	0.065	−0.048	**0.030**	0.635	0.065	−0.057	0.073	0.220
Th5	**0.046**	−0.044	**0.030**	0.147	0.058	−0.062	0.149	0.147	**0.005**	−0.165	**0.018**	**0.016**
Th6	**0.041**	−0.054	**0.010**	0.147	0.130	−0.051	**0.018**	0.313	0.108	−0.094	0.073	0.056
Th7	0.080	−0.069	**0.010**	0.713	0.274	−0.084	**0.003**	0.562	0.185	−0.101	**0.030**	0.492
Th8	0.413	−0.030	**0.010**	0.635	0.118	−0.080	**0.010**	0.713	0.170	−0.071	**0.018**	0.428
Th9	0.294	−0.052	0.432	0.220	0.142	−0.063	**0.010**	0.635	0.525	−0.050	0.149	0.958
Th10	0.856	0.015	0.530	0.635	**0.004**	−0.086	**0.005**	0.056	0.413	−0.057	0.202	0.713
Th11	1.000	−0.010	0.202	0.220	0.413	−0.040	0.876	0.492	0.892	−0.039	1.000	0.492
Th12	0.525	0.036	0.073	0.635	0.080	−0.036	1.000	**0.031**	0.201	−0.048	0.755	0.118
L1	1.000	−0.003	0.755	0.635	0.440	−0.042	0.432	0.313	0.413	−0.055	0.876	0.220
L2	0.440	0.027	1.000	0.428	0.964	−0.019	0.343	0.492	0.586	−0.086	0.876	0.147
L3	0.856	−0.023	0.876	0.368	0.892	−0.012	0.876	1.000	0.170	−0.044	0.432	0.056
L4	0.235	−0.027	0.149	0.562	1.000	−0.026	0.202	0.492	0.316	−0.046	0.432	0.263
	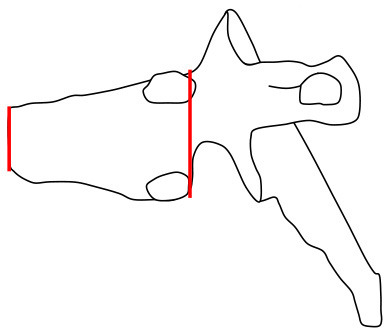				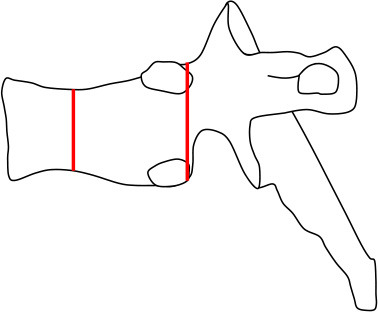				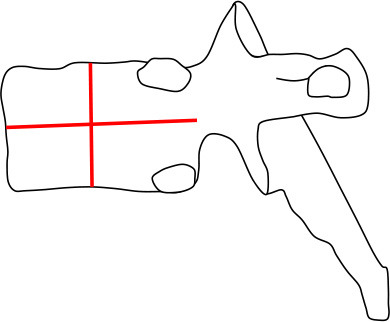			

p-values calculated with Mann–Whitney U test; values <0.05 considered significant and marked in bold. The 15 mutation-positive subjects harbor different PLS3 mutations: nine with a heterozygous/hemizygous deletion c.73–24T>A (p.Asp25Alafs*17), three with heterozygous/hemizygous intragenic tandem duplication within PLS3, one with heterozygous missense mutation c.1424A>G (p.Asn446Ser), and two with heterozygous/hemizygous nonsense mutation c.766C>T (p.Arg256*). Th, thoracic vertebra; L, lumbar vertebra; AP, anterior–posterior; MP, mutation-positive; MN, mutation-negative; M, male; F, female.

Overall appearance of the vertebrae was similar in both mutation-positive and -negative subjects. Isolated findings of heterogenous coloring or intravertebral adiposity were noted. One female subject (MP14) had one large hemangioma in L4 (Figure 2).

#### Vertebral Endplates

**Figure 6:**
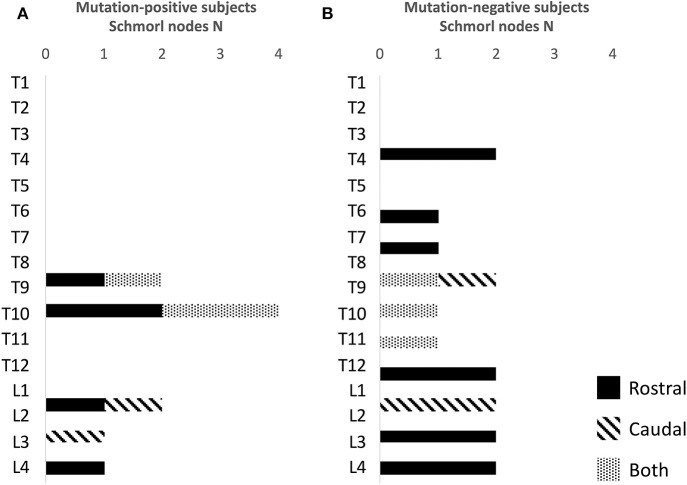
Number and location of Schmorl nodes in **(A)** 15 *PLS3* mutation-positive and **(B)** 13 mutation-negative subjects. The *PLS3* mutation-positive subjects harbor different mutations: nine with heterozygous/hemizygous deletion c.73–24T>A (p.Asp25Alafs*17), three with heterozygous/hemizygous intragenic tandem duplication within *PLS3*, one with heterozygous missense mutation c.1424A>G (p.Asn446Ser), and two with heterozygous/hemizygous nonsense mutation c.766C>T (p.Arg256*).

For both mutation-positive and -negative subjects, SNs were most prevalent in the lower thoracic and lumbar spine. Both groups had both rostral and caudal SNs and the total number was similar in both groups [5/15 (33%) vs. 4/13 (31%)]. The range and total number of all SNs was higher in the mutation-negative subjects: 1–2 vs. 1–10 and 10 vs. 16, respectively (Figure 6).

#### Intervertebral Discs

Although vertebral height reduction was significant and the vertebrae most often biconcave in shape in the mutation-positive subjects, no obvious changes were observed in the intervertebral discs. Median surface areas of the intervertebral discs from L3 to L5 were 913 and 933 mm^2^ (*p* = 0.943). Some intervertebral discs were enlarged and balloon-shaped secondary to the biconcavity of the abnormal shape of vertebrae (Figures 2B–D).

#### Spinal Alignment

**Figure 7:**
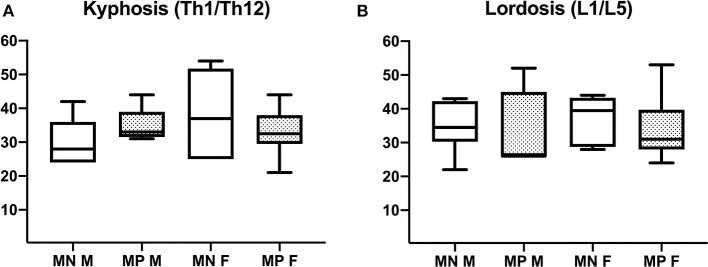
Comparison of **(A)** thoracic kyphosis and **(B)** lumbar lordosis in 15 *PLS3* mutation-positive and 13 mutation-negative subjects. Thoracic kyphosis was measured from T1 to T12 and lumbar lordosis from L1 to L5. MP, mutation-positive; MN, mutation-negative; M, male, F, female. The *PLS3* mutation-positive subjects harbor different mutations: nine with heterozygous/hemizygous deletion c.73–24T>A (p.Asp25Alafs*17), three with heterozygous/hemizygous intragenic tandem duplication within *PLS3*, one with heterozygous missense mutation c.1424A>G (p.Asn446Ser), and two with heterozygous/hemizygous non-sense mutation c.766C>T (p.Arg256*).

VCFs caused changes in the mutation-positive subjects' spinal alignment that was most often visually evident as a notch in the upper thoracic spine. Although abnormal vertebrae were prevalent in the mutation-positive subjects, secondary enlargement of intervertebral discs tended to maintain the spinal alignment. Thus, the overall degree of thoracic kyphosis was similar when calculated with the Cobb's method: 28 vs. 33° for the mutation-positive and -negative subjects, respectively (*p* = 0.950) (Figure 7). Due to the thoracic notch, the degree of lordosis was decreased, and the lumbar spines appeared straighter than in the mutation-negative subjects. The change in lumbar lordosis was more pronounced than change in kyphosis, although statistically not significant; the medians for lordosis were 37 vs. 30° for the mutation-positive and -negative subjects (*p* = 0.520) (Figure 7).

## Discussion

This study is the first to report a systematic review of spinal MRI findings in a large cohort of patients with defective PLS3 function. We assessed the spine in 15 mutation-positive subjects with different *PLS3* mutations in four unrelated Finnish families. Results were compared with congruent findings in 13 mutation-negative individuals from the same families. We have previously described that mutations in the X-chromosomally inherited *PLS3* lead to a skeletal disorder predominantly in males characterized by compromised bone strength, low rate of bone formation, defective mineralization, and consequently prevalent fractures and loss of adult height (5, 8, 12). Findings in the present study indicate that defective PLS3 function leads to severe age- and sex-related abnormalities in vertebral morphology already in early childhood and to significant spinal pathology by early adulthood. In contrast, spinal cartilaginous structures show no premature deterioration.

The changes in vertebrae were significantly more severe and prevalent in our cohort of *PLS3* mutation-positive subjects compared with the mutation-negative subjects. Despite the relatively small number of subjects, reductions in anterior or mid-vertebral heights were significantly greater as compared with mutation-negative subjects, and most common in upper thoracic vertebrae (T3–T8). These changes were also prevalent even in children and young adults; significant anterior height reduction was present in T6 and mid-vertebral height in T10 for subjects under 30 years of age. Although no longitudinal data were available for individual subjects, the changes also had an apparent progressive course with increasing age as the older subjects presented with most widespread and severe spinal deformities. When compared with mutation-negative subjects, males were predominantly more severely affected with a greater difference in mid-vertebral height reduction, specifically in T4 and T6–T10. Though an anticipated sex difference was seen, the females also had marked vertebral changes, while the level of statistical significance was reached only in T12. Morphological classification indicated that most often the changes were compression deformities resulting in vertebral biconcavity.

We have previously reported severe spinal pathology in a cohort of subjects with another form of primary osteoporosis caused by a heterozygous loss-of-function mutation in *WNT1* (14). The 17 *WNT1* mutation-positive subjects exhibited prevalent VCFs predominantly in the lower thoracic spine, consequently exaggerated thoracic kyphosis and loss of adult height. No VCFs were apparent in children and only came apparent after the age of 50 years and seemed to progress with increasing age thereafter (14). In comparison, our current study demonstrates that *PLS3* mutations seem to introduce vertebral changes that are different in shape and spinal location, appear at a much younger age and show greater magnification with increasing age (Figure 2).

In addition to skeletal changes, we evaluated possible cartilaginous tissue deterioration. Although PLS3 is ubiquitously expressed in several other tissues, no constant extra-skeletal manifestations are associated with PLS3 osteoporosis (4, 5, 8, 18). Congruently, *Pls3*-KO mice exhibit no evident extra-skeletal abnormalities (11). Complete absence of Pls3 in a zebrafish model resulted in embryonic lethality (11, 19). Tsolis et al. reported high expression of PLS3 in chondrocytes isolated from osteoarthritis (OA) patients' articular cartilage, proposing a role for PLS3 in chondrocyte regulation, while chondrocyte maturation appeared normal in *Pls3*-KO mice (11, 20). Besides these, no detailed data on PLS3 function in chondrogenesis is available. On the other hand, studies on spinal muscular atrophy have evidenced that PLS3 partakes in formation of neuromuscular synapses and that its overexpression can salvage axonal defects in a SMA mouse model (18, 21). Individual PLS3 patients have been reported to have muscle hypotonia (8), but no systematic review of muscle involvement in PLS3 osteoporosis has been reported.

In the present study the spinal cartilaginous tissues were assessed by evaluating defects in vertebral endplates and intervertebral discs. The number of SNs was similar in both groups and even slightly higher in the mutation-negative subjects. Further, vertebral endplates were well-preserved despite the grave vertebral deformities. SNs represent intravertebral disc herniations arising from disc degeneration and weakened vertebral bone and are present even in the healthy population (22). In contrast, we have previously reported increased prevalence of SNs, enlarged intervertebral discs and endplate deterioration in WNT1 osteoporosis, which we hypothesized to arise from aberrant WNT pathway activation and its consequent impact on the coupled osteogenesis and chondrogenesis (14). From those results, we deduced that spinal cartilaginous deterioration is a distinct feature of WNT1 osteoporosis and SNs, since prevalent also in children, could possibly predict future VCFs. In contrast, cartilaginous tissue deterioration does not seem to be a typical characteristic of PLS3 osteoporosis and the difference to WNT1 osteoporosis is evident when comparing the spinal structures and stature in *PLS3* and *WNT1* mutation-positive subjects (Figure 2). Interestingly, while the effect of the *WNT1* mutation seems to be harmful for cartilaginous tissues in vertebral endplates, we have previously reported that *WNT1* mutation-positive subjects show less age-related deterioration in knee articular cartilage indicating that in articular cartilage the effect of the *WNT1* mutation could have protective and preservative consequences instead (23). The impact of *PLS3* mutations on articular cartilage outside the vertebral endplates has not be systematically studied. In light of the previously reported increase in PLS3 expression in OA patients' articular chondrocytes indicating a role for PLS3 in OA pathogenesis (20), it could also be postulated that decreased PLS3 function, as in the case of loss-of-function *PLS3* mutations, could have cartilage-preserving effects.

Our study has some limitations, primarily concerning the relatively small cohort size, slightly unequal sex and age distribution in the study groups and the cross-sectional nature of the study. Also, the use of multiple MR-imaging machines and some subjects' prior or on-going osteoporosis medication can be considered as confounding factors. The MR-images were taken in supine position and compared against data taken in standing position. Several studies have indicated that kyphosis is decreased in supine vs. standing position (24) and hence this study is likely to underestimate the degree of kyphosis or lordosis. Also, the relatively small cohort size posed limitations to statistical analyses and may have prevented us from observing some relevant differences between the groups. Regardless of these limitations, we consider our study setting to be unique with the largest-to-date cohort and individuals from the same families, blinded nature of examinations, and good inter-reader reliability. We therefore consider our findings novel and of great value given the rarity of *PLS3* mutation-positive subjects and the lack of knowledge of PLS3 function in bone and its relative skeletal pathology. These offer novel and clinically valuable information about the differences between various forms of primary osteoporosis, providing tools for clinical differential diagnosis.

We conclude that defective PLS3 function due to pathogenic variants in *PLS3* causes significant, early-onset and sex-related pathology in the axial skeleton both in males and females. Changes in vertebral shape and height, presence of vertebral compression fractures and consequent alterations in spinal alignment are prevalent already in childhood and become increasingly more severe and numerous affecting multiple vertebrae with age. Changes in spinal cartilaginous tissues are not present. Timely diagnosis and intervention are key in alleviating progression of these pathological changes and reducing pain, physical limitations and impaired quality of life. Regardless of the growing number of reported *PLS3* mutation-positive individuals and clinical data on PLS3-related bone diseases, our current knowledge on the function of PLS3 in bone and other tissues is limited and our current means of diagnosis and treatment insufficient. Further studies in larger cohorts are needed to attain added information and help generate targeted treatments.

## Data Availability Statement

All datasets generated for this study are included in the article/supplementary material.

## Ethics Statement

The studies involving human participants were reviewed and approved by Research Ethics Board of Helsinki University Hospital. Written informed consent to participate in this study was provided by each participant or their legal guardian/next of kin.

## Author Contributions

RM, TN, JN, and OM: study design. RM, TN, JN, MS-P, ST-S, and OM: study conduct. RM, TN, AK, AC, and JN: data collection. RM, TN, JN, MS-P, and ST-S: data analysis. RM: drafting manuscript. RM and OM take responsibility for the integrity of the data. All authors: revising manuscript content and approving final version of the manuscript.

## Conflict of Interest

The authors declare that the research was conducted in the absence of any commercial or financial relationships that could be construed as a potential conflict of interest.
